# Patient perspectives of jail based MOUD treatment: views of individuals who have returned to the community following incarceration

**DOI:** 10.1186/s40352-025-00319-7

**Published:** 2025-04-22

**Authors:** Claudia Santelices, Warren Ferguson, Rebecca Rottapel, Ekaterina Pivovarova, Elizabeth Evans, Thomas Stopka, Peter Friedmann

**Affiliations:** 1 Northeastern University, Boston, USA; 2 Department of Family Medicine and Community Health, University of Massachusetts Chan Medical School, Worcester, MA USA; 3 Department of Public Health and Community Medicine, Tufts University School of Medicine, Boston, MA USA; 4 Department of Health Promotion and Policy, School of Public Health and Health Sciences, University of Massachusetts, Amherst, MA USA

**Keywords:** Jails, Opioid use disorder, Medication for opioid use disorder, MOUD implementation, EPIS framework, qualitative methods

## Abstract

**Background:**

Massachusetts passed legislation in 2018 to mandate provision of medications for opioid use disorder (MOUD) in select jails to address the high risk of opioid overdose after release. Since 2019, we have conducted a Type-1 hybrid effectiveness-implementation study of this program. We present findings on the perspectives and experiences of persons treated in these jails.

**Methods:**

We conducted qualitative analyses of semi-structured interviews that were conducted in 2022 with 38 adults released to the community who had been treated with MOUD while living in one of eight Massachusetts jails. The Exploration, Preparation, Implementation, and Sustainment (EPIS) framework informed development of the interview guide and analysis of qualitative data. Deductive and inductive strategies were used for coding and analyses.

**Results:**

Participants were 41.5 years old; predominantly male; 84.2% white, 23.7% Hispanic/Latino and 7.9% Black; and most continued taking MOUD. Thematic analysis focused on four code reports: Perception of Addiction/MOUD, Barriers/Facilitators, Impact of MOUD in Jails, and Stigma. Participants perceived that MOUD helped to prevent relapse. Prompt and consistent access to medication, and respectful treatment by healthcare and carceral staff were highlighted as facilitators. In contrast, some participants perceived that policy-centered rather than patient-centered treatment drove timing of medication initiation or response to medication changes. Insufficient staffing and the COVID-19 pandemic contributed to treatment delays. Overall, individuals incarcerated in jails that have expanded treatment eligibility to include earlier induction with MOUD generally felt more positive about their experience than individuals reporting delayed induction.

**Conclusions:**

Participants valued the ability of jail based MOUD programs to help clients achieve recovery from OUD. Their perceptions highlight the intrinsic value of MOUD programs that promote and support wellbeing through a person-centered approach to treatment. Participants stressed that MOUD programs should be patient-centered and guided by patients’ symptoms and needs.

**Supplementary Information:**

The online version contains supplementary material available at 10.1186/s40352-025-00319-7.

## Background

For over two decades, opioid overdoses have taken a devastating toll in communities across the U.S., and limited access to effective medications for opioid use disorder (MOUD) has been a major contributor to this crisis (CDC, 2018). Massachusetts has been one of the most severely affected states, with mortality rates quintupling from 5.9 per 100,000 in 2000 to 33.6 per 100,000 in 2022, and more than 2,000 opioid-related overdose deaths per year between 2016 and 2022 (Massachusetts Department of Public Health, 2023). Ample evidence indicates that individuals experiencing incarceration are at a much higher risk of overdose and premature death following release compared to the general population. (National Academies of Sciences, Engineering, and Medicine, 2019; Winkelman et al., 2018; Binswanger et al., 2007; Ranapurwala et al., 2018).

To address this opioid crisis affecting justice involved individuals, in 2017 the Commission on Combating Drug Addiction and the Opioid Crisis recommended the use of Medication for Opioid Use Disorder (MOUD) with pretrial detainees and its continued use upon release from incarceration. Subsequently, in 2022, the U.S. Department of Justice (DOJ) released guidance to acknowledge and enforce OUD as an instance of the American Disabilities Act (ADA), indicating that people with OUD have a disability because their drug dependency substantially limits one or more of their major life activities, therefore they must be protected when using legally prescribed MOUD (U.S. Department of Justice 2022).

Until 2019, few jails and prisons in the U.S. provided incarcerated individuals with Food and Drug Administration (FDA)-approved MOUD. Massachusetts was one of the first states to pioneer efforts to reduce the impact of the opioid overdose epidemic with a state legislative mandate (Chap. 208, Massachusetts General Laws, 2018) to expand access to all FDA-approved MOUDs (extended-release naltrexone [XR-NTX], buprenorphine-naloxone [BUP-NX], methadone, and more recently, Brixadi (buprenorphine) extended-release injection for subcutaneous use), and to facilitate MOUD post-release coordination of care in a selected number of county correctional facilities (Houses of Corrections and jails, heretofore referred to as jails). Chapter 208 established a 4-year pilot program to expand all FDA-approved forms of MOUD at five county jails, with two additional jails joining in the initiative in 2019. Additional county jails began to offer MOUD in subsequent years. The law stipulates that MOUD be maintained for individuals receiving it prior to their detention and initiated prior to release among sentenced individuals whenever deemed clinically appropriate (Massachusetts General Laws, 2018). In addition, the U.S. Dept of Justice issued guidance regarding ensuring access to MUD in jails and prisons (US Dept of Justice, 2022).

Since 2019, the Massachusetts Justice Community Opioid Innovation Network (MassJCOIN) partnered with participating jails and community treatment providers to conduct a Type 1a multi-method hybrid effectiveness implementation study of Chap. 208 (NIDA UG1DA050067; mPIs: Friedmann and Evans). This initiative has important implications for future policy and practice in the justice and OUD treatment systems at local, state, and national levels, and it offers a valuable opportunity for a naturalistic study to develop understanding on MOUD program outcomes, implementation, and cost (Evans et al., 2021). We present preliminary findings on the perspectives and experiences of formerly incarcerated individuals treated for OUD in eight Massachusetts county jails. This manuscript provides the patient perspective, and as such, it incorporates views of justice-involved individuals regarding opioid use disorder and MOUD treatment in jail settings whose perspectives have not been included in similar research. We anticipate that our findings will provide valuable information to improve MOUD programs or assist in subsequent design, implementation, and advocacy that responds to lived experiences of incarcerated individuals (Hoffman et al., 2023).

## Methods

### Study site and participant recruitment

Eligible participants received an FDA-approved MOUD while incarcerated after Sept 1, 2019, in one of eight county jails in Massachusetts offering MOUD, and were subsequently released. The counties spanned the entire state, with varying levels of population density (rural and urban). Recruitment was conducted via flyers distributed strategically in locations known to be frequented by previously incarcerated individuals (jail release packets, community treatment services, transitional housing, etc.). In addition, study staff attempted to contact potential participants who previously provided permission to the jail to be contacted post-release.

### Data collection

Semi-structured interviews, along with brief demographic surveys, were conducted by telephone from 2021 to 2022. All participants provided verbal informed consent prior to study enrollment and were compensated with $40. Interviews were digitally recorded and transcribed. Transcripts were reviewed for accuracy and clarity. Interviewers were female and male and included a social worker, anthropologist, epidemiologist, clinical psychologist, public health PhD candidate, and master-level staff members; all had prior experience conducting qualitative research interviews (Evans et al. 2023). Research procedures incorporated COREQ criteria (Tong A, 2007). All research procedures were approved by the Baystate Health Institutional Review Board.

**Fig. 1:**
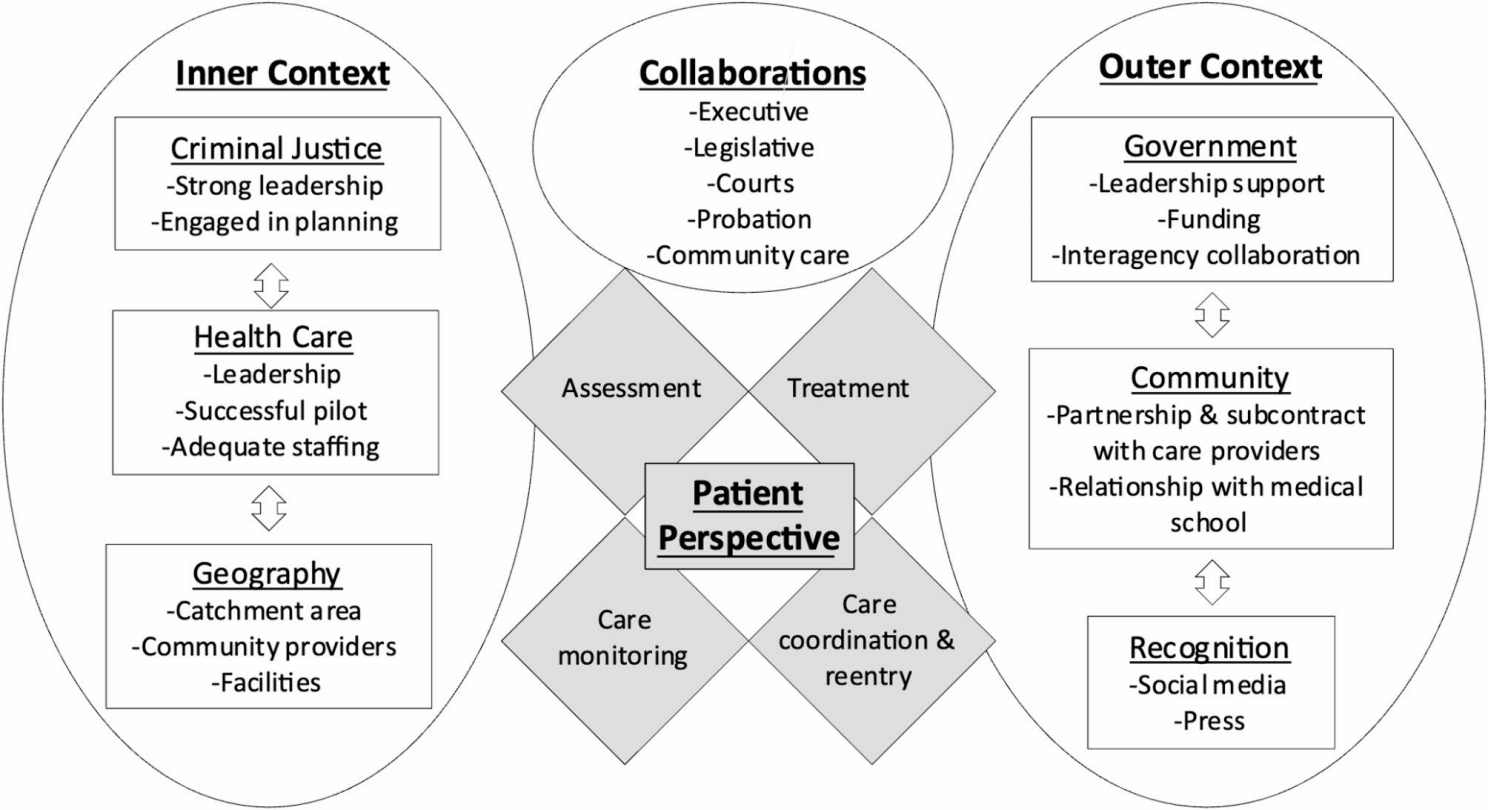
Exploration, preparation, implementation, sustainment (EPIS) framework Adapted from Aarons et al., 2011

An implementation science framework for public service programs, EPIS (Exploration, Preparation, Implementation, Sustainment), informed development of the interview guide (Aarons et al., 2011). Employment of this framework and modifications which incorporate MOUD treatment processes (Fig. 1) has been well described in other published manuscripts by our team. (Ferguson et al., 2019; Evans et al., 2021; Evans et al. 2023) Our interview guide was designed to better understand patient experiences while incarcerated (Implementation) and their suggestions for improvement (Implementation and Sustainment) (Interview guide appended in Appendix 1). For the present paper, we analyzed data elicited by participants’ responses to prompts related to perceptions of addiction and recovery broadly (including attitudes, knowledge, and beliefs), perceptions about MOUD treatment generally and treatment received in the jails (including positive and negative opinions related to efficacy and the role of medication in SUD treatment), and personal recommendations for improvement and sustainability of MOUD treatment programs in jails.

### Data analysis

Data analysis utilized deductive and inductive strategies. The parent study developed a codebook using a priori codes based on the interview guide designed to address study aims. Emerging codes were refined using open coding and iterative comparative methods, resulting in a codebook with 23 parent codes and 32 child codes (for details, see Evans et al., 2023). Subsequently, four staff formed two dyads. Dyad members coded each transcript independently and met to check for consistency in coding. Discrepancies were discussed with the entire team until agreement across the two dyads was achieved. Codes were entered into Dedoose, version 9.0.107 (https://www.dedoose.com, 2021). Next, we identified patterns in coded data, comparing salient themes and organizing them into overarching domains (Glaser & Strauss 2017). We examined patterns within and across transcripts and grouped similar responses with illustrative quotations. The current manuscript presents findings stemming from the analysis of four codes: Perception of Addiction/MOUD, Perceived Barriers/Facilitators throughout Implementation, Impact of HOC MOUD, and Stigma.

## Results

Participants had a mean age of 41.5 years and were predominantly male (85.7%). Most participants were White (84.2%), with 23.7% self-identifying as Hispanic/Latino persons and 7.9% as Black persons. Most participants were taking MOUD (95%) at the time of the interview (Table 1).

**Table 1:** Client interview participant characteristics (*n* = 38)

Characteristic	Count (%)
**Age**, mean (SD)	41.5 (9.3) Missing = 1
**Female**, n (%)	4 (14.3%)
**Race**,** Hispanic/Latino Ethnicity** n (%)	
Native Hawaiian or Other Pacific Islander Black or African American White More than one race	1 (2.6%) 3 (7.9%) 32 (84.2%) 2 (5.3%)
**Hispanic/Latino ethnicity**,** n (%)**	9 (23.7%)
**Education**, n (%)	
No high school diploma High school diploma or equivalent Some college, but no degree Associate’s degree	8 (21.1%) 19 (50.0%) 9 (23.7%) 2 (5.3%)
**Not currently taking MOUD**, n (%) **Currently taking MOUD**, n (%) Buprenorphine (e.g., Suboxone, Subutex) XR-Bup (Sublocade) XR-Naltrexone (Vivitrol) Methadone	2 (5.3%) 36 (94.7%) 20 (55.6%) 4 (11.1%) 1 (2.8%) 11 (30.6%)

Our data underscore how participants value the ability of MOUD program to help clients achieve recovery from substance use and recovery of health and social functioning. *Two main Implementation themes emerged from data analyses: (a) general perspectives on jail based MOUD programs and challenges of treating addiction; and (b) personal experiences (both positive and negative) with treatment during the most recent incarceration.*

### Theme 1: general perspectives on jail based MOUD programs and challenges of treating OUD

#### Theme 1.1: MOUD is necessary but not sufficient

According to all participants, MOUD programs must be a core therapeutic component in every jail because they can assist individuals pharmacologically to prevent withdrawal symptoms and return to drug use. Yet consensus exists among some participants that FDA-approved medications, whether used inside or outside carceral settings, cannot guarantee a “cure” or long-lasting abstinence without therapeutic components such as individual and group counselling.

“I make it my goal to learn something new every day. So, when I was there, I liked the counseling process, and I also liked the group process, because you actually hear and learn things that you would never even thought was part of the process. So, in my perspective, it was helpful, because not only-, not only in the addiction process aspects of it, but also in the mental health aspects of it.” [ID101].

Participants expressed that while behavior driven by opioid addiction is an individual choice, it is shaped by the presence or absence of multiple individual, community, and social factors, as is engaging in MOUD treatment. When reflecting on the importance of jail based MOUD programs, most participants stated that it is imperative that program staff see the value of therapeutic treatment while acknowledging and addressing the complex contours of opioid addiction and the monumental challenges involved in seeking recovery. Jail-based MOUD programs that do so, and are invested in addressing both, are rendered essential to have hope in regaining one’s life. Two main aspects of recovery that characterized some of our participants’ experiences, and that transcended the strictly pharmacological domain, were the need for aspirational goals, and the presence of community resources upon release. First, recovery could be achieved and sustained in the presence of aspirational goals, or something to look up to during or after incarceration. As one participant said,

“I have goals and aspirations, and in order to meet my goals and have the life I want– you know, I have to participate in my recovery and everything else that goes along with it. I’m going to fight tooth and nail for my recovery because my life is worth it, and my recovery is worth it, and I love my family and I don’t want to live that life anymore… So, I believe if – I believe the tools are there if somebody really truly wants it” [ID 202].

Second, OUD is a disease and a symptom of deeper and larger social ills. Indeed, some participants emphasized that accessibility to support services could help prevent a return to drug use and even decrease overdose rates following release. In the absence of said resources, drugs could be sought to alleviate the pain of deprivation. “People sometimes use drugs to numb everything”, said one participant [ID 306], and another added:

“…… In jail, some people have everything, but then when they get out, they have nothing, nobody, you know what I mean? They’re miserable. So, all they want to do is just go get high, so they don’t feel the pain…” [ID 601].

Responses shared by participants indicated that two main reasons make jail-based MOUD programs attractive or beneficial to justice-involved individuals suffering from OUD: treatment prepares individuals for the outside world, and jail-based programs replicate environments and relations existing outside the carceral premise. We address these, in turn.

#### Theme 1.2: treatment prepares for the outside world

First and foremost, participants valued MOUD programs in carceral settings because initiating treatment in jail and coordination and continuation post-release helps to reduce risk of overdose and prepares people to manage their lives. As one participant stated:

“Yeah. I think it’s a great thing to have because then you set people up where when they get out of jail, they already have a routine. They send them off. When you leave jail, they already set you up with your prescriptions or with a clinic to go to, so you just stay on that track of just getting healthy and eventually you can move down and get off it until you get back on your feet so you could just function. It’s such a great thing to have, and I think it’s an awesome idea. I think every jail should do it.” [*ID 403*].

The reentry programming in jail based MOUD programs highlighted in the quote above is one of participants’ main motivations for program enrollment and adherence before they reenter the community.

#### Theme 1.3: MOUD programs create therapeutic environments in jail

According to some participants, delivery of MOUD treatment helps jails reproduce treatment environments in the outside world to which they will return once released. For example, in a carceral setting, correctional officers and service providers will be expected to engage in respectful behavior, referring to MOUD programs as treatment, without mediating stigmatizing behaviors. As one participant described:

“I believe it was a non-judging - I think, they [correctional and treatment staff] believed you. I think, they thought MAT was good. I mean, some people have their thing on it. Like, “Oh, you’re not sober because you’re on MAT,” but I think that’s bull crap. Because I’m saying I’m going on five months sober.” [ID 403].

Along these lines, MOUD programs in jails must address both pharmacological unmet needs while incarcerated and following release, as well as help reduce the stigma associated with the medication itself. In other words, jail-based MOUD programs must also help those with or without OUD reframe MOUD as a “medicine” and not “a drug”. In one participant’s words, changing the lens through which MOUD in jails are perceived, could yield the following positive outcome:

“This treatment is good because they [MOUD providers] are thinking about human beings, too. There are many people who are hooked on it on the street, and I know they have been arrested, but they [MOUD providers] are there, helping, helping them [incarcerated individuals] to get off drugs. Because since Suboxone is a drug too, but that takes away the cravings, and that is a controlled substance. It is not like other drugs; it is a medicine. …” [ID 303].

### Theme 2: personal experiences in treatment programs

Findings on patient experiences with the jail based MOUD program focus on care processes and are depicted in a modified version of the EPIS framework displayed in Fig. 1. This manuscript focuses on three of the four treatment processes, assessment, treatment, and care monitoring during incarceration, and suggestions for improvement going forward. We anticipate an additional manuscript focused on the fourth program process, care management and reentry. Participants’ experiences, both positive and negative, resulted in a set of patients’ recommendations on program implementation going forward. These recommendations, presented below, either highlight the need to reinforce program features perceived as beneficial or change policies and procedures to improve treatment outcomes.

#### Theme 2.1. staff treat clients as “human beings”: communication and relationships with staff

**Fig. 2:**
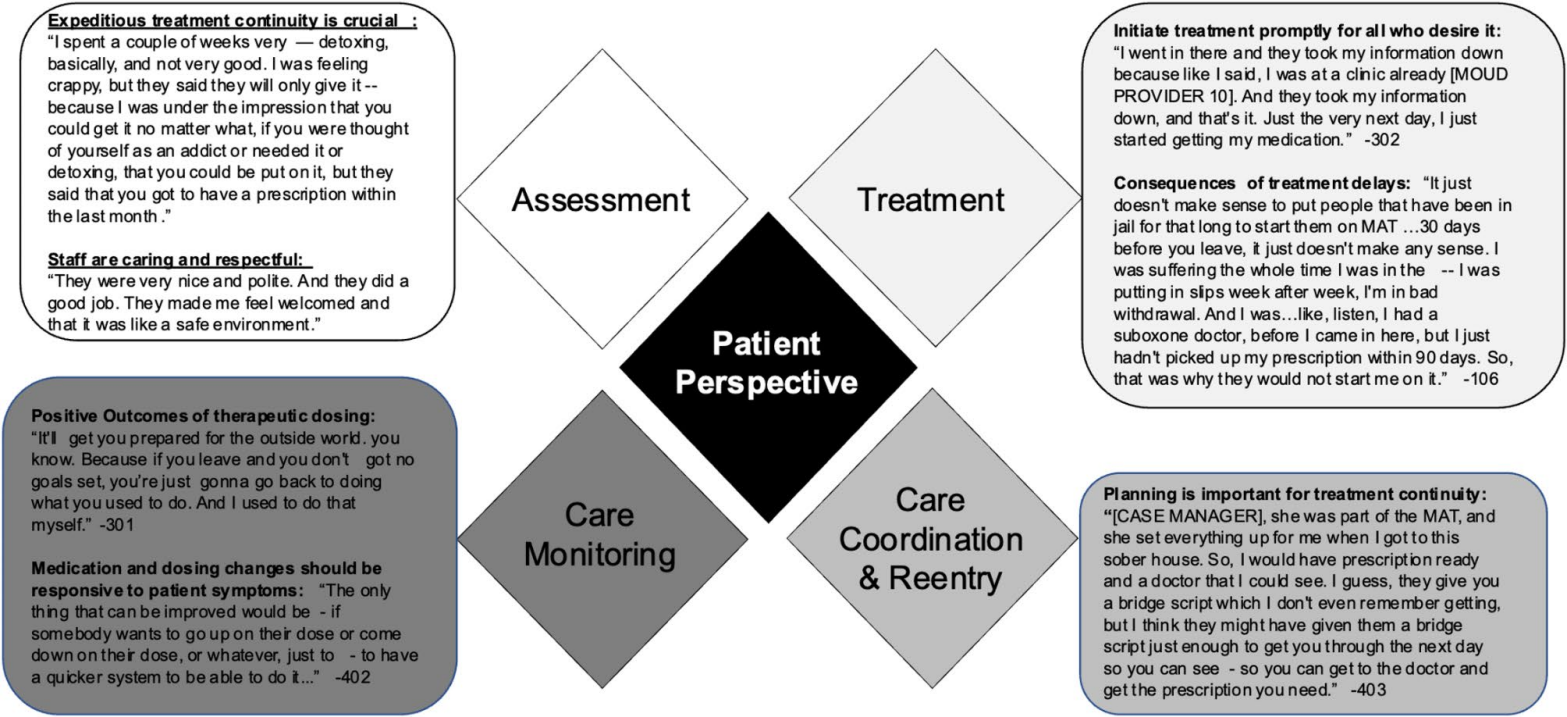
Patient perspectives on jail based MOUD programs

Transcending all care processes highlighted in Fig. 2 is the quality of communication and relationships with staff, which participants described as “caring”, “respectful, “supportive”, and “empathizing”. According to our participants, contrary to the culture usually experienced in carceral settings, these caring behaviors signaled treatment of incarcerated persons as human beings, an unequivocally welcome change. According to most participants, these qualities facilitate effective communication and could contribute to achieving desired treatment outcomes.

“I couldn’t ask for a better support. I just—I never thought that I would have care like that, you know what I mean? Like actual people who gave a shit about you, (chuckles) you know what I mean? Like, who were, you know, concerned, considerate, and made sure that you were—everything that you needed was taken care of.” [ID 502].

This respectful treatment extended to security staff involved with the program. As the quote below illustrates, in some cases, correctional officers were perceived as contributing to creating and reinforcing a therapeutic, respectful, and comforting environment.

“Correctional officers, well a lot of them, will talk down to you, you know, they’ll treat you different. When it came to the conventional teaching [on treatment] they’ll, you know, treat you with respect. They were more like connected with us. They wanted to help us you know. They made us feel comfortable and they didn’t talk down to us.” [ID 301].

Other participants, however, noted variation in the degree of respect or disrespect from correctional officers as well as other incarcerated persons. Manifestations of disrespect often reinforced the stigma that opioid medications are nothing but the equivalent to “dope,” and incarcerated individuals receiving them are nothing but “junkies.”

“It varied. Some of them [correctional or clinical staff] would joke like, ‘Yeah, go get your dope. We’re going to give them dope to get high,’ versus others who were, didn’t care about it one way or another. It was just another program in the jail and then, there were others. There are few that I think cared at least acted like they did. So, I think it was split up, you know, all different ranges”. [ID 603]

Disrespect from incarcerated peers was also reported highlighting stigma towards OUD and individuals receiving MOUD.

“You had other inmates that would call you junkies, losers, you know, you had people that just talked shit about you because you was using the medication.” [ID 301].

#### Theme 2.2: patient assessment and decision to treat

Assessment defines the process for deciding whether a patient meets criteria for continuation or initiation of treatment at the time of jail entry. The legislation articulated circumstances requiring treatment, which was translated into policy and procedures at each jail. All jails followed these requirements, but some have expanded treatment initiation to any person with OUD who desires treatment.

##### Theme 2.2.1 expeditious assessment to begin MOUD dosing upon entry to jails is crucial

Participants who experienced prompt dosing at the time of entry described it as an important contributor to their stability. On the contrary, those who did not receive prompt dosing expressed the stress they experienced during the waiting period.

“I mean if you’re – you come into jail and you’re taking methadone or say I’m on my dose in the streets and I come in and I don’t – I’m not dosed and I’m sick, I could relapse. Do you know what I mean? Stuff like that. And like… And like not only that it’s just – it’s become such a disability and like a stable thing in my life. It’s part of a routine that I’ve settled into and like to break that, to completely disrupt like the recovery that I have, you know…” [ID 304].

In other words, jail-based MOUD programs can avoid treatment gaps by promptly assessing patients and reinstating the treatment routine individuals had prior to being incarcerated. And, as the following section highlights, some participants pointed to the timeliness and reliability of dosing as important components of the success of jail-based programs. Many participants were highly complementary about the treatment they received.

“I went in there and they took my information down because like I said, I was at a clinic already, [MOUD PROVIDER 10], and they took my information down, and that’s it. Just the very next day, I just started getting my medication.” [ID 302].

Most of the constructive criticism centered on the policies and practices around treatment enrollment. An overarching recommendation was to treat OUD as an illness and provide symptom-based treatment rather than following predetermined policies and procedures. Challenges with withdrawal experiences permeated these recommendations.

“Because they said I didn’t have a script within the last month that they would not give it to me. And so I spent a couple of weeks very— detoxing, basically, and not very good, I was feeling crappy, but they said they will only give it -- because I was under the impression that you could get it no matter what, if you were thought of yourself as an addict or needed it or detoxing, that you could be put on it, but they said that you got to have a prescription within the last month. So, it took me some fighting, and some complaining, and the fact that I was going to a program, which then they decided to finally give it to me after a couple of weeks” [ID 602].

Some participants took issue specifically with policies stating that treatment induction can only begin 30 days before release for those who were not in treatment at the time of intake.

“It just doesn’t make sense to put people that have been in jail for that long to start them on MAT all of a sudden or not like 30 to 90 days, it’s usually like around 30 days before you leave, because if you’re going to prison they’re having people that are lifers that have been sober for years, or whatever, people are getting out all of a sudden, and like, the people are for 10 years, whatever. And you’re starting them a month before they leave. It’s like, it just doesn’t make any sense. I was suffering the whole time I was in the -- I was putting in slips week after week, I’m in bad withdrawal. And I was even like, to the point where I’m like, listen, I had a suboxone doctor, before I came in here, but I just hadn’t picked up my prescription within 90 days. So, that was why they would not start me on it.” [ID 106].

Participants did consider myriad reasons for delays in treatment initiation beyond actual policies when this was explained to them. High demand during periods of low staffing, especially during the COVID pandemic was an issue. “[T]hey were really understaffed in the MAT department over there” [ID 106]. This personnel issue was also true for entry into jail on weekends when staffing was lower. In addition, participants noted that staff reported concern for causing precipitation of opioid withdrawal if treatment was started too early, especially for those using fentanyl, the predominant opioid available for purchase on the street.

“So, it took me I think seven days to like get on it. I believe it was something like that because they were afraid of me getting sick because of the heroin. That’s one thing. I mean, I’m not sure about the whole thing, but I know after at least three days and you’re pretty much good.” [ID 403].

##### Theme 2.2.2: better patient education and communication on treatment options

Participants reported some variation in the quality of patient education/information about treatment availability, options and procedures, and some inconsistencies in the timeliness of the delivery of such education/information to newly incarcerated individuals. One person reported a high quality of education but noted that it was inconsistent and that transparency about the treatment plan would be helpful. The need for clarity and uniformity in treatment education is clearly addressed in the following excerpt:

“I think that they should talk to them more at intake, you know, of what the process is. I think, you know, you get into the infirmary and you’re kind of left in the jail cell with five, six other girls or men, and no one’s really talking to you, and you know that you have a script and or you don’t even know what the process is. … most people really don’t wanna talk to the nurse at intake, if they’re detoxing but at least give them a somewhat of an idea of like, you’re gonna be talking to a provider within the next 24 hours, whatever the case may be. For me, it was fast, for some people it wasn’t. So, I don’t know what the criteria is for that to be one way or the other” [ID 701].

Some participants also emphasized the need for incarcerated MOUD programs to assure the availability of all FDA approved medications as mandated, however, this was not always the case.

“It seems like Suboxone was kind of what they were easily putting people on, but you couldn’t get on methadone when you’re in there. And if you didn’t have, so yeah, they kind of forced you to be put on Suboxone. And they offer any actual detox, there’s no taper there’s nothing like so. [*And did you bring that up to the clinical staff at some point or not*,* it was just pretty much to yourself? ]* Yeah, I brought that up many times and so did other people, and then like depending on some time, sometimes they would come down and they would take some people, and then you never know when you were coming back, and we’d have to write a slip to ask them a question and they would never, they would just never respond to it.” [ID 403].

As the following interview excerpt highlights, other participants noted that while incarcerated Vivitrol was not even described as an option for treatment, but they became aware of it once released into the community.

“I was going to say just that would be an option in the future too. I mean if they offered that I’m sure some people would do that too. *[So*,* you weren’t aware of Vivitrol at all? ]* No, not in jail. I mean, I know about it afterwards, but yeah.” [ID403].

#### Theme 2.3 treatment

Participants also spoke about the day-to-day experiences of participants being in treatment with MOUD and how treatment was integrated into their daily routine.

##### Timing of treatment

For participants, consistency in the daily timing of treatment determined their confidence in the program and had a positive impact on how they conducted their lives while incarcerated. As illustrated in the excerpts below, staff at times went the extra mile to be proactive and diligent to ensure adherence to treatment:

“… [Dosing] was regular; general ballpark time in the morning they give it to you if you were at a program school they would come and find you. You never really had to worry about getting dosed. Saw the doctor really regular, once a month or so. So yeah, I had really no gripes about it. I think everything went pretty good.” [ID 603].

On the contrary, other participants noted that variation in dosing time is disruptive to recovery and daily functioning.

“So, we get up, and it happens at all different times of the morning or the day, you never know exactly when you’re gonna go, it’s usually within about like a four-hour window. You might go right away, or you might wait for hours, so you never go at the same time, and that’s an issue.” [ID602].

One participant also noted that separate med lines help to facilitate efficient MOUD dosing.

“We were separated from the other people, during med call. We were preferenced above other people in med line.” [ID204].

##### Housing policies for those in treatment

There were some differences of opinion regarding housing policies. Some participants saw that mixing housing for MOUD patients with the general population was good in that it normalized OUD. Others saw segregation of the treatment population from regular counterparts as facilitating a more efficient administration of the medication. Both viewpoints are illustrated in the excerpts below:

“They had us mixed in with general population. They didn’t have like a unit for you know, people that was on MAT. They didn’t have us all in one block, so we would spread out… I think it’s good because eventually everybody’s gonna have to get used to MAT participants being around them. I mean, you know, it’s a real problem in a community, it’s going to be a problem in a facility. You know, you got all walks of life in there.” [ID301].

“If they have certain units for the guys that are on the MAT program you can just go to that unit and distribute it to everybody in a proper way. Instead of having one unit come down and wait till they finish and go back up to their unit to bring the other unit down and that’s what I call a shit show.” [ID705].

#### Theme 2.4 care monitoring

Care monitoring encompasses ongoing monitoring of MOUD treatment, including treatment effectiveness, addressing potential side effects and response to patient requests for medication or dosing changes.

##### Medication and dosing changes should be responsive to patient symptoms

Most participants emphasize the importance of having a MOUD program that addresses patients’ concerns in a timely manner with respect to type of medication or dose received. Contrary to this, some participants reported that requests for dose changes or changing to a different medication were often ignored.

“So, I would continuously put in requests to get a dose increase because I felt like it wasn’t doing anything for me, the dose that I was on. I would still experience cravings and withdrawal and stuff like that. So, I didn’t – I wasn’t getting my necessary, you know, the necessary effects of the reason why I chose the medication to be on in the first place. I wasn’t receiving any of that. So, I kept asking for increases…”[ID504].

“The only thing that can be improved would be, I would say somebody like - if somebody wants to go up on their dose or come down on their dose, or whatever, just to - to have a quicker system to be able to do it.”[ID402].

#### Overall impact of MOUD treatment program in jails reported by participants

Participants in this study are clear on the benefits of MOUD treatment in the sense that the way in which it is implemented acknowledges what they think is the meaning of recovery or what this encompasses, a reduction/cessation of substance use as well as the recovery of functioning in other health and social domains.

“The big payoff was that I didn’t have to come off my canteen, or I didn’t have to call and lie and say I need money for this and it was really for that.” [ID 301].

“So, once your head clear, you’re already down yourself enough, so it’s good to just have that motivation to whatever, get your highest at, your GED or do schooling, to participate in the programs, the family programs, to learn how to be a better father and all that.” [ID 403].

“It’ll [MOUD] get you prepared for the outside world you know. Because if you leave and you don’t got no goals set, you’re just gonna go back to doing what you used to do. And I used to do that myself.” [ID 301].

## Discussion

We document the perspectives of formerly incarcerated persons who participated in MOUD treatment with all FDA approved medications in eight jails. Ours findings stem from participants’ general perceptions on “addiction” and recovery, and their personal experiences during their most recent incarceration. They add to the published research over the last three years and complement the rich tapestry of voices from different actors involved in the MOUD program, from clinical to correctional staff, community MOUD providers to jail leaders (Matsumoto et al., 2022; Stopka et al., 2022; Pivovarova et al., 2022; Evans et al., 2022; Evans et al., 2023). This is one of the first efforts to advance knowledge via feedback from treatment participants, and it is our hope that this work informs the implementation of evidenced-based treatment with MOUD and any attempt to initiate or improve MOUD treatment.

### General perceptions

When asked about their general perceptions of jail based MOUD programs participants highlighted the intrinsic value of treatments that address the complexities of opioid use disorder through pragmatic contributions to individuals’ overall wellbeing. As such, their insights on the value of MOUD programs are consistent with current consensus statements on the concept of recovery as a process that encompasses change in multiple domains of health, purpose, and community (SAMHSA, 2022). Thus, in their view, MOUD treatments must facilitate full participation in rehabilitative programming and preparation to succeed when reentering non-carceral communities. These programs must provide uninterrupted access to all FDA- approved medications for OUD for those already in recovery; access to psychoeducational therapeutic treatment, and various forms of socio-economic support to help reduce risk of overdose and prepare individuals to manage their lives upon release.

Apart from the above-mentioned programmatic components, some participants also valued MOUD programs that invest in both, the quality of *the service* being delivered and the quality in the *service delivery*. Findings suggest that jail based MOUD programs should attempt to replicate therapeutic environments and relationships existing outside the carceral premise and that are not mediated by stigmatizing behaviors. In other words, addressing opioid use disorders in carceral settings must involve bridging actions to see the human behind the addict and the medicine behind the “drug”.

### Personal experiences

The analyzed data on participants’ experiences before, during, and after receiving MOUD in jail underscore the urgency of jail based MOUD treatment and the need to maximize positive treatment outcomes during and after release. In addition to treatment availability, participants highlighted the importance of a well-articulated care process where assessment, care monitoring and care coordination upon community reentry are anchored by the human qualities of MOUD providers. Participants noted the respect, caring attitudes, and empathy of staff, which ran counter to their previous expectations. These experiences reduced engrained stigma due to substance use disorders, mental illness, and criminal-legal involvement (Atkins et al., 2020; Volkov ND, 2020; Schnittker, J., & John, 2007). Participants were also insightful about operational elements that contributed to the success of treatment including timely assessment and qualification for treatment, efficient initiation of treatment, standardized treatment procedures such as timing of medication administration and strategies such as separate medication lines.

Not surprisingly, given that MOUD treatment programs including agonist medications are relatively new, participants reported some variation in program policies and operations. Massachusetts Chap. 208 law requires continuation of treatment for those documented to have been on community-based treatment at the time of facility entrance, and mandates treatment induction for those with OUD 30-days prior to release, and care coordination for seamless continuation of community-based treatment following release. The most frequent criticisms focus on how each facility interpreted the law and determined patient eligibility. Participants cited that a lapse in filling a prescription in the community prior to arrest or an inability to document treatment at the time of entry disqualified them for needed treatment in jail. Such individuals experienced painful opioid withdrawal syndrome. Other reasons for delays in treatment continuation or initiation included low staffing, especially during the pandemic and on weekends. Many participants reported understanding that the COVID-19 pandemic created extraordinary challenges for starting and sustaining MOUD treatment in jails. Others cited delays for fear of precipitating withdrawal from fentanyl. However, several participants failed to understand the reasons for delays in response to requests to change their medication dose or type. In nearly all these circumstances, participants suggested that symptoms and need should drive decisions for MOUD treatment.

This study’s focus on patient’s experiences of MOUD implementation in jails is novel. However, studies on implementation of other evidence-based treatment have incorporated patient experience to improve outcomes. For example, human-centered design (HCD) has been employed in studies on HIV treatment to improve outcomes in communities (Beres et al., 2019). Patient-reported outcome and experience measures have proven to facilitate improvements in the design of evidence-based interventions (Stover et al., 2021). Moreover, understanding patient experiences can ground the differing perspectives of treatment providers, carceral staff and leaders engaged in the implementation of MOUD treatment. Not surprisingly, patients also point to variation in the implementation of treatment from site-to-site, a phenomenon common to rollout of new treatment programs (Benzer et al., 2013).

Translating legally mandated MOUD treatment into successful programming in carceral settings has been an enormous undertaking, especially given the historic pandemic that emerged soon after program initiation. Moreover, creating effective policies and procedures and advancing change management is challenging. Thus, it is not surprising that early participants, while often complimentary of the effort, took seriously our interest in understanding how to improve the programs. The most important constructive criticisms were leveled at rigid policies that adhered to the “letter of the law” rather than the urgent needs of patients. These needs include starting medication for anyone needing treatment and providing timely and ongoing evaluation for participants experiencing side effects or inadequate treatment responses and who request changes in the prescribed medication or its dosage. We are hopeful that these findings will inform the writing of future regulations and amendment to current laws and their translation to policies.

## Limitations

This study has limitations. First, all but one of the recruited subjects have continued with treatment. Thus, findings are reflective of persons who have succeeded in treatment and may not represent the experiences of individuals who have stopped treatment following release. Nonetheless, participant observations included both positive and negative attributes of their treatment experiences. Second, despite efforts to recruit a diverse group of participants, the participants interviewed for the study were largely male and White. Few participants identified as Hispanic or Black, and only one participant identified as female. Although participants were from a limited geographic region, namely Massachusetts, they did come from both urban and rural communities. Finally, in some circumstances, a particular theme did not reach data saturation, but the data presented was sufficiently compelling and relevant to implementation to warrant inclusion of the theme. For example, while only one individual noted that a delay in treatment transpired due to concerns about overdose precipitation due to fentanyl, it was an important contribution, especially considering recent research results suggesting that acute overdose with early treatment is a rare phenomenon (D’Onofrio et al. 2023).

## Conclusions

Despite these limitations, the findings on the patient perspective contribute to our understanding of how best to implement MOUD in carceral settings. This analysis of patient experiences with MOUD treatment programs in county jails in Massachusetts reveals strong support for the programs from participants. Beyond treatment availability, participants reported more respectful treatment by both clinical and carceral staff which further contributed to their success with initiating or continuing with recovery. Participants recognized that opioid use disorder is a lifelong chronic disease coupled with serious morbidity and mortality. While noting many program successes, participants emphasized that treatment should be patient-centered and guided by symptoms and needs, rather than rigid policies and procedures.

## Electronic supplementary material

Below is the link to the electronic supplementary material.

Supplementary Material 1

Supplementary Material 2: Appendix 1

## Data Availability

No datasets were generated or analysed during the current study.

## References

[CR1] Aarons, G. A., Hurlburt, M., & Horwitz, S. M. (2011). Advancing a conceptual model of evidence-based practice implementation in public service sectors. *Administration and Policy in Mental Health*, *38*(1), 4–23. 10.1007/s10488-010-0327-7 21197565 10.1007/s10488-010-0327-7 PMC3025110

[CR2] Atkins, J., Dopp, A. L., & Temaner, E. B. (2020). Combatting the stigma of addiction - The need for a comprehensive health system approach. NAM Perspect. ; 2020:10.31478/202011d 10.31478/202011d PMC8916801 35291745

[CR3] Benzer, J. K., Beehler, S., Cramer, I. E., et al. (2013). Between and within-site variation in qualitative implementation research. *Implementation Sci*, *8*, 4. 10.1186/1748-5908-8-4 10.1186/1748-5908-8-4 PMC3598511 23286552

[CR4] Beres, L. K., Simbeza, S., Holmes, C. B., Mwamba, C., Mukamba, N., Sharma, A., Munamunungu, V., Mwachande, M., Sikombe, K., Bolton Moore, C., Mody, A., Koyuncu, A., Christopoulos, K., Jere, L., Pry, J., Ehrenkranz, P. D., Budden, A., Geng, E., & Sikazwe, I. (2019). Human-centered design lessons for implementation science: Improving the implementation of a patient-centered care intervention. *Journal of Acquired Immune Deficiency Syndromes*, *82*(3), S230–S243. 10.1097/QAI.0000000000002216 31764259 10.1097/QAI.0000000000002216 PMC6880397

[CR5] Binswanger, I. A., Stern, M. F., Deyo, R. A., Heagerty, P. J., Cheadle, A., Elmore, J. G., & Koepsell, T. D. (2007). Release from prison–a high risk of death for former inmates. N Engl J Med. ;356(2):157–65. 10.1056/NEJMsa064115. Erratum in: N Engl J Med. 2007;356(5):536. 10.1056/NEJMsa064115 PMC2836121 17215533

[CR6] Center for Disease Control and Prevention. Understanding the epidemic. Centers for Disease Control and Prevention (2018). Retrieved from https://www.fraudfighters.net/wp-content/uploads/2021/03/Understanding-the-Epidemic-_-Drug-Overdose-_-CDC-Injury-Center.pdf. Accessed [date].

[CR10] D’Onofrio, G., Hawk, K. F., Perrone, J., Walsh, S. L., Lofwall, M. R., Fiellin, D. A., & Herring, A. (2023). Incidence of precipitated withdrawal during a multisite emergency department-initiated buprenorphine clinical trial in the era of fentanyl. *JAMA Netw Open*, *6*(3), e236108. 10.1001/jamanetworkopen.2023.6108 36995717 10.1001/jamanetworkopen.2023.6108 PMC10064247

[CR8] Data Brief: Opioid-Related Overdose Deaths among Massachusetts residents (2023). Retrieved from:https://www.mass.gov/doc/opioid-related-overdose-deaths-among-ma-residents-december-2023/download. Accessed: January 11, 2024.

[CR9] Dedoose Version 9.0.107. (2021). *Dedoose Version 9.0.107 cloud application for managing, analyzing, and presenting qualitative and mixed method research data*. SocioCultural Research Consultants, LLC. https://www.dedoose.com

[CR12] Evans, E. A., Stopka, T. J., Pivovarova, E., Murphy, S. M., Taxman, F. S., Ferguson, W. J., Bernson, D., Santelices, C., McCollister, K. E., Hoskinson, R. Jr, Lincoln, T., Friedmann, P. D., & MassJCOIN Research Group. (2021). Massachusetts justice community opioid innovation network (MassJCOIN). *Journal of Substance Abuse Treatment*, *128*, 108275. 10.1016/j.jsat.2021.108275 33483222 10.1016/j.jsat.2021.108275 PMC8263807

[CR11] Evans, E. A., Pivovarova, E., Stopka, T. J., Santelices, C., Ferguson, W. J., & Friedmann, P. D. (2022). Uncommon and preventable: Perceptions of diversion of medication for opioid use disorder in jail. *Journal of Substance Abuse Treatment*, *138*, 108746. 10.1016/j.jsat.2022.108746 35249789 10.1016/j.jsat.2022.108746 PMC9167208

[CR40] Evans, E. A., Pivovarova, E., Senthilkumar, R., Rottapel, R. E., Stopka, T. J., Santelices, C., Ferguson, W. J., Friedmann, P. D. (2023). Diversion of medications to treat opioid use disorder: Qualitative findings from formerly incarcerated adults in Massachusetts. Int J Drug Policy. 122:104252. PMID: 37980776. 10.1016/j.drugpo.2023.104252 PMC10841635 37980776

[CR13] Ferguson, W. J., Johnston, J., Clarke, J. G., Koutoujian, P. J., Mauer, K., Gallagher, C., White, J., Nickl, D., & Taxman, F. S. (2019). Advancing the implementation and sustainment of medication assisted treatment for opioid use disorder in prisons and jails. *Health and Justice*, *7*, 19. 10.1186/s40352-019-0100-2 31832801 10.1186/s40352-019-0100-2 PMC6908545

[CR14] Glaser, B., & Strauss, A. (2017). *Discovery of grounded theory: Strategies for qualitative research*. Routledge.

[CR15] Hoffman, K. A., Thompson, E., Gaeta Gazzola, M., Oberleitner, L. M., Eller, A., Madden, L. M., Marcus, R., Oberleitner, D. E., Beitel, M., & Barry, D. T. (2023). Just fighting for my life to stay alive: A qualitative investigation of barriers and facilitators to community re-entry among people with opioid use disorder and incarceration histories. *Addict Sci Clin Pract*, *18*, 16. 10.1186/s13722-023-00377-y. Published online 2023 Mar 21. 36944998 10.1186/s13722-023-00377-y PMC10031976

[CR27] Massachusetts General Laws. (2018). An Act for Prevention and Access to Appropriate Care and Treatment of Addiction, Chapter 208, Section 98. https://malegislature.gov/Laws/SessionLaws/Acts/2018/Chapter208

[CR28] Massachusetts Department of Public Health. (2023). Data Brief: Opioid-Related Overdose Deaths among Massachusetts residents. Retrieved from: https://www.mass.gov/doc/opioid-related-overdose-deaths-among-ma-residents-december-2023/download. Accessed: January 11, 2024.

[CR16] Matsumoto, A., Santelices, C., Evans, E. A., Pivovarova, E., Stopka, T. J., Ferguson, W. J., & Friedmann, P. D. (2022). Jail-based reentry programming to support continued treatment with medications for opioid use disorder: Qualitative perspectives and experiences among jail staff in Massachusetts. *International Journal of Drug Policy*, *109*, 103823. 10.1016/j.drugpo.2022.103823 35994938 10.1016/j.drugpo.2022.103823 PMC10206716

[CR17] National Academies of Sciences, Engineering, and Medicine. (2019). In A. I. Leshner, & M. Mancher (Eds.), *Medications for opioid Use Disorder Save lives* (p. 25310). National Academies. 10.17226/25310 30896911

[CR29] Pivovarova, E., Evans, E. A., Stopka, T. J., Santelices, C., Ferguson, W. J., & Friedmann, P. D. (2022). Legislatively mandated implementation of medications for opioid use disorders in jails: A qualitative study of clinical, correctional, and jail administrator perspectives. Drug and Alcohol Dependence, Article 109394. 10.1016/j.drugalcdep.2022.109394 10.1016/j.drugalcdep.2022.109394 PMC9169252 35349918

[CR18] Ranapurwala, S. I., Shanahan, M. E., Alexandridis, A. A., Proescholdbell, S. K., Naumann, R. B., Edwards, D. Jr, & Marshall, S. W. (2018). Opioid overdose mortality among former North Carolina inmates: 2000–2015. *American Journal of Public Health*, *108*(9), 1207–1213. 10.2105/AJPH.2018.304514 30024795 10.2105/AJPH.2018.304514 PMC6085027

[CR19] Substance Abuse and Mental Health Services Administration. (SAMHSA, 2022) Recovery and Recovery Support, 2022. Retrieved from: https://www.samhsa.gov/find-help/recovery. Accessed February 14, 2024.

[CR20] Schnittker, J., & John, A. (2007). Enduring stigma: the long-term effects of incarceration on health. J Health Soc Behav. ;48(2):115–30. 10.1177/002214650704800202. PMID: 17583269. 10.1177/002214650704800202 17583269

[CR21] Stopka, T. J., Rottapel, R. E., Ferguson, W. J., Pivovarova, E., Toro-Mejias, L. D., Friedmann, P. D., & Evans, E. A. (2022). Medication for opioid use disorder treatment continuity post-release from jail: A qualitative study with community-based treatment providers. *International Journal of Drug Policy*, *110*, 103803. 10.1016/j.drugpo.2022.103803 35965159 10.1016/j.drugpo.2022.103803 PMC10117037

[CR22] Stover, A. M., Haverman, L., van Oers, H. A., Greenhalgh, J., Potter, C. M., & ISOQOL PROMs/PREMs in Clinical Practice Implementation Science Work Group. (2021). Using an implementation science approach to implement and evaluate patient-reported outcome measures (PROM) initiatives in routine care settings. *Quality of Life Research*, *30*(11), 3015–3033. 10.1007/s11136-020-02564-9 32651805 10.1007/s11136-020-02564-9 PMC8528754

[CR23] Tong, A., Sainsbury, P., & Craig, J. (2007). Consolidated criteria for reporting qualitative research (COREQ): A 32-item checklist for interviews and focus groups. *International Journal for Quality in Health Care*, *19*(6), 349–357. 10.1093/intqhc/mzm042 17872937 10.1093/intqhc/mzm042

[CR24] U.S. Department of Justice (2022). The Americans with disabilities act and the opioid crisis: Combatting discrimination against people in treatment or recovery. Civil Rights Division. Available at: https://archive.ada.gov/opioid_guidance.pdf Last accessed January 17, 2025.

[CR25] Volkow, N. D. (2020). Stigma and the toll of addiction. *New England Journal of Medicine*, *382*(14), 1289–1290. 10.1056/NEJMp1917360 32242351 10.1056/NEJMp1917360

[CR26] Winkelman, T. N. A., Chang, V. W., Binswanger, I. A., & Health (2018). Polysubstance Use, and criminal justice involvement among adults with varying levels of opioid use. *JAMA Netw Open*, *1*(3), e180558. 10.1001/jamanetworkopen.2018.0558 30646016 10.1001/jamanetworkopen.2018.0558 PMC6324297

