# Supplementary material for: Patient perspectives of jail based MOUD treatment: views of individuals who have returned to the community following incarceration
Source: Health Justice. 2025 Apr 22;13:25. doi: 10.1186/s40352-025-00319-7 (PMC12016191; doi:10.1186/s40352-025-00319-7)
Supplement: Supplementary file 1 — Supplementary Material 1 [file 40352_2025_319_MOESM1_ESM.pdf]

Table: Result Themes and Sub-Themes

Theme 1: General perspectives on jail-based MOUD programs and treatment challenges

1.1 MOUD is necessary but not sufficient

1.2 Treatment prepares for the outside world

1.3 MOUD programs create therapeutic environments in jail

Theme 2: Personal experiences in treatment programs

2.1 Staff treat clients as "human beings": communication and relationships with staff

2.2 Patient assessment and decision to treat

- 2.2.1. Expeditious assessment to begin MOUD dosing
- 2.2.2 Better patient eduction and communication on treatment options

2.3 Treatment

- 2.3.1 Timing of treatment
- 2.3.2 Housing policies for those in treatment

2.4 Care monitoring

- 2.4.1 Medication and dosing changes should be responsive to patient symptoms

2.5 Overall impact of MOUD treatment program in jails reported by participants
