# Supplementary material for: Patient perspectives of jail based MOUD treatment: views of individuals who have returned to the community following incarceration
Source: Health Justice. 2025 Apr 22;13:25. doi: 10.1186/s40352-025-00319-7 (PMC12016191; doi:10.1186/s40352-025-00319-7)
Supplement: Supplementary file 2 — Supplementary Material 2: Appendix 1 [file 40352_2025_319_MOESM2_ESM.docx]

**Appendix 1. Semi-Structured In-Depth Interview Guide**

**Introduction**

*Hello. My name is ____ and I am part of the research team at ______. I want to talk with you about the program to provide medications to treat opioid use disorder to people in jails in Massachusetts. Your experiences, thoughts, and suggestions will help to improve the program. Thank you for talking with me!*

*As a reminder, your participation in the study is completely voluntary. You can decline to answer any question. You can stop the discussion at any time and for any reason.*

***[if applicable]*** *I will record our conversation. That let me focus on the conversation and not miss a word of what you say. Let me know if at any time you decide you do not want to be recorded or if you’d like me to stop the recorder.*

*After the interview, we will transcribe the [recording/notes] to analyze the responses. We will not include your name or identifying information in the final transcribed interviews.*

*When I talk about “medication treatment” I mean any methadone, buprenorphine (suboxone, subutex, etc), or vivitrol (naltrexone) to treat opioid use disorder that you received in jail or after release in the community. Do you have any questions before we start?*

*[****interviewer:*** *if participant has had several jail incarcerations,* ***ask about the most recent incarceration*** *in which they received medications for opioid use disorder]*

**SECTION I: WHILE IN JAIL**

1. Where were you incarcerated? For how long? When were you released?
2. Did you come into the jail already on medication treatment (prescribed only)?

| If yes… | If no… |
| --- | --- |
| Which one?  What did you have to do to start or continue your medication treatment in jail? | How did you decide which type of medication to take in jail?  What medication did you get?  Did you request this med, were you given options? How did that come about?  Were there **any differences** between the clinician and you with regard to which medication to put you on? How were those differences handled?  Tell me about how the process for starting the medication worked…  Did you experience any dosing issues? If so, what was that like for you? (Suggested rewording: Talk me through the process to receiving your medication, starting with being in the unit).  Probe: For example, did you want to receive a higher or lower dosage amount than what was provided? If yes, tell me more. |

1. What was your overall experience with receiving medication to treat opioid use disorder in jail? How did you feel about that medication?
2. What types of meds were available to treat your opioid use disorder (in jail)? What was availability like for each medication? Were you given the opportunity to choose your medication? Did you feel the jail encouraged use of a certain medication over the other(s)?
3. What are some things that worked well in receiving medication treatment in jail (e.g., dosing, appointment, time from appt to receipt of medication, requests to change initial treatment plan)?
4. Based on your experience at [Sheriff Office/Dept NAME], what are some things that could be improved in receiving medication treatment at this jail?
5. Were there times when a patient on medication had it stopped? What happened when someone started medication treatment and then stopped it?
6. Have you ever received injectable, long-acting medication for opioid use disorder?

| If yes… | If no… |
| --- | --- |
| - Which one? What was your experience like?   - Probe about the one never received: What have you heard about injectable naltrexone (Vivitrol)// injectable buprenorphine (Sublocade or Brixadi)? - If a monthly or weekly injection of buprenorphine (the main ingredient in Suboxone) were available in the jail, would you consider it? Why or why not? | - What have you heard about injectable naltrexone (Vivitrol) or injectable buprenorphine (Sublocade or Brixadi)? - If a monthly or weekly injection of buprenorphine (the main ingredient in Suboxone) were available in the jail, would you consider it? Why or why not? |

1. What, if any, experience did you have with counseling or other talk therapy as part of the treatment program in the jail?
   1. How did it go?
   2. What, if any, requirements were there for participation?
   3. Who runs these programs?
   4. Did you like these programs? Any concerns about timing, frequencies, etc.
   5. What other services did you receive for your opioid problem, if any? Were you satisfied with those services? Why, or why not?
   6. Was there another service you were expecting to receive but didn’t receive? Explain.
2. What do you think correctional or clinical staff thought about medication treatment? What were your interactions with them like? What were the opinions of other staff and inmates about some people receiving medication treatment?
3. Were patients receiving medication treatment treated differently than other inmates by correctional or clinical staff? In what ways? (example probe: did it impact where people were housed?)

**SECTION II: RE-ENTRY INTO THE COMMUNITY**

1. Regarding your medication for OUD while incarcerated, what was the dosage amount that you were on when you were released from jail? *(just looking for numerical response):* ___________________
2. While in jail, did anyone talk to you about continuing medication treatment in the community?

| If yes… | If no… |
| --- | --- |
| - Who asked? What was that like? - How did the jail help you to continue medication treatment post-release? Was this helpful? How can it be improved? - Probes: activation of Medicaid or other insurance, get a valid ID, bridge scripts, make treatment appointments, transportation, housing, social support. | - Do you know why a plan was not discussed? |

1. Did you continue medication treatment after release?

| If yes… | If no… |
| --- | --- |
| - What are some of the things you had to do to continue treatment? - Which medication are you being prescribed now? - [IF DIFFERENT MEDICATION THAN IN JAIL] Why are you on a different medication? (e.g., own decision, prescribed by doctor without participant’s consent) - Was there any gap in your receipt of the medication since you left jail? Why? **How did that affect you?** | - Why did you not get medication treatment in the community after release? (travel, insurance, medication didn’t work, schedule) - What could have been different so that you could have continued medication after jail? - What would need to happen for you to go back to medication treatment? |

1. (if taking medications now) what is the dosage amount that you are on now? *(just looking for numerical response):* _____20mg______________

**SECTION III: WRAP-UP**

1. What are some of the main things that explain why people do or do not overdose after release from jail or prison??
   1. *Probe*: Is this based on your experience or what you see in others?
   2. *Probe*: “Main things” tied to overdose could include…
      1. Personal background and characteristics (e.g., age, gender, race/ethnicity, identity; addiction severity; medical or mental health conditions)
      2. Social or community connections and context (e.g., family, friends, running buddies; social stigma related to addiction or MOUD; social support for treatment or recovery etc.)
      3. Institutional experiences (e.g., shelters, soup kitchens, specific corrections facilities, drug treatment programs, etc.)
   3. *Probe*: Tell me about some things that other people may not have thought about that could make people more or less likely to overdose.

[IF THERE IS TIME] Let’s go back to your experience while you were in jail.

1. What stories, if any, did you hear about people on medication treatment in jail diverting their dose or doing other things to give their medications to others?
2. How did the medication treatment program impact the flow of illegal buprenorphine or other contraband substances into the jail? Did the price of illegal buprenorphine or other substances change as a result of the program?
3. How did having access to medication treatment in the jail impact your use of opioids?
4. What are some lessons that you learned that can be useful for other jails implementing medication treatment?
5. Did the COVID-19 pandemic change your experience of the jail medication treatment program? Has COVID-19 affected your ability to get medication treatment in the community after release? How?

We have talked about many things today. Is there anything else that you feel I should know or that we haven’t covered but you feel is important for me to know?

Conclusion: Thank you so much for talking with me today – I really appreciate it. If you have any concerns, please don’t hesitate to reach out! Thanks again!
